# Socio-economic inequalities in health-related quality of life among Iranian young people in the middle stage of adolescence: application of Health Equity Assessment Toolkit

**DOI:** 10.1186/s12887-022-03815-z

**Published:** 2023-01-11

**Authors:** Azam Maleki, Elham Faghihzadeh, Samaneh Youseflu, Shahnaz Zamani barjasteh

**Affiliations:** 1 Social Determinants of Health Research Center, Zanjan University of Medical Sciences, Zanjan, Iran; 2 Department of Epidemiology and Biostatistics, School of Medicine, Zanjan University of Medical Sciences, Zanjan, Iran; 3 Department of midwifery and reproductive health, Isfahan University of Medical Sciences, Isfahan, Iran; 4 Zanjan University of Medical Sciences, Zanjan, Iran

**Keywords:** Health-related quality of life, Socio-economic, Inequality, Adolescence

## Abstract

**Background:**

One of the main concerns of public health is the increasing inequality of health status, which has an adverse effect on people’s life.

**Purpose:**

The current study aims to analyze the role of socioeconomic inequalities in health-related quality of life (QoL) among Iranian young people in the middle stage of adolescence.

**Methods:**

A cross-sectional descriptive study was conducted on 576 young people in the middle stage of adolescence. The samples were selected using the Multi-stage sampling method. Data were collected by a demographic checklist, and KIDSCREEN-52 questionnaire and analyzed by SPSS ver.16. The Health Equity Assessment Toolkit (HEAT) Version 4.0 (beta) was used to assess adolescents’ QoL inequalities in terms of socio-economic subgroups.

**Results:**

The results show that 27.2 adolescents had low quality of life. The score of physical and autonomy components of QoL was significantly more in male versus school environment in female adolescents. Also, the asset index, father’s, and mother’s education, and family income in female adolescents, and the assets and family income in male adolescents were significantly related to the quality of life (*p* < 0.05). The risk of lower QOL in the poorest quintile was 1.12 times more than in the richest quintile. The consideration index of Asset in terms of sex was 4.5 and the modified Gini index was more than 0.5 in females and males.

**Conclusion:**

Our findings highlight the significant effects of socioeconomic inequality on the HRQL of Iranian adolescents. Requires a targeted policy approach to reach the poorest quintile for improving the quality of life of adolescents.

## Introduction

Adolescence as a transition period is one of the most critical, and sensitive stages of life for adolescents and their families. Upon entering this course of life, adolescents experience profound physical, psychological, social, and cognitive changes, so it is very important to pay attention to their health and well-being of them from different aspects [1, 2]. Furthermore, their health and well-being are essential for their growth and development, including the acquisition of emotional and cognitive abilities for independence, completion of education, transition to employment, civic participation, and the establishment of lifelong relationships [1–3]. As a result, childhood-living conditions are considered the basis, and foundations of lifelong health [4]. In some counties, little investment has been made in adolescent health needs, and their concerns in the primary health care system are neglected [5]. In Iran, like in other countries, due to quarantine conditions and social distance, some families have experienced changes in terms of social, cultural, economic, and lifestyle [6]. Providing a clear and complete overview of the situation and the health inequality could help to prioritize the target audience’s needs and technical knowledge [7].

Assessing youth and adolescents’ quality of life is of great importance due to the rapid emotional and cognitive changes that are characteristic of this age group [5, 8]. Health-Related Quality of Life (HRQL) is a measure used to assess physical and social functioning, mental health, and well-being as health outcomes among children and adolescents, as well as to evaluate population-based intervention programs [8]. The components of adolescents ‘quality of life are different from those of adults because, in addition to the physical and psychological components, their relationship with family and peers and the school environment should also be considered, which is less important in adulthood [9]. Therefore, the quality of life of adolescents should be considered in different dimensions. Measuring quality of life can improve clinical decision making, evaluate the quality of medical care, estimate health care needs in a particular population, understand the different causes and consequences in health, and ultimately health policy [10, 11]. Some experts consider the quality of life of children and adolescents as a mental and changeable feeling about their health and believe that this feeling is a reflection of their desires, hopes, and expectations about their current and future life situation, which is influenced by factors such as gender, age, personal and family characteristics, as well as economic and social status [12, 13].

Socioeconomic status (SES) indicated an overall measure of the economic and social status of people or their family relative to others, which were measured by income, occupation, and education [14]. The socio-economic status and family environment are related to an adolescent health condition, so unhealthy habits, unavailability of healthcare services, and face higher socio-economic pressure are more seen in people who have low SES [5, 15]. Furthermore, the availability of material and social resources and reactions to stress-inducing conditions by peers, and their family can impress adolescent health [15, 16]. Also, SES is related to health inequality. There is an enormous body of evidence showing the effect of S on children and adolescents’ quality of life [5, 12, 16, 17]. The adolescence QoL could be influenced by many factors, such as SES, parental educational level, better work status, household income, age, gender, higher family wealth, high level of social capital, migration, positive family climate, personal factor (i.e., self-efficacy, optimism, sense of coherence), and social support [5, 12, 16, 17].

The previous research highlighted the effect of SES on various aspects of child and adolescent health. In this regard, Rajmil et al. study emphasize the role of SES in the mental health of children and adolescents aged 8–18 years according to the family level of education in 11 European countries [18]. Sfreddo et al. show a higher level of oral QoL in adolescence with lower mean income school’s neighborhood, household income, and maternal schooling [19]. Moreover, the Iranian cohort study indicates a significant association between SES and school functioning, psychosocial function, and QoL [20].

The problem of poverty and low SES cannot be investigated without considering the way of the income distribution in this society [21]. While the concept of poverty is related to the lowest class in the classification of the income distribution, inequality in income distribution is related to all classes of society [21]. There is considerable evidence that has emphasized the effect of social class gradients on the health and mortality of children and adolescents [20, 22]. Also, the socio-economic inequality (SEI) measures are considered a reflection of the levels of justice and fairness in society [21]. Although inequalities exist across different demographic groups and communities based on gender, race or ethnicity, age, periods, geographic region, and health status, socio-economic status is considered an important factor in these differences [21]. One of the main concerns of public health is the increasing inequality of socio-economic factors, which has an adverse effect on people’s quality of life. In this regards Niedzwiedz et al. study show that peoples in more generous welfare regimes experienced narrower socioeconomic inequalities and higher level of QoL [23]. Also, there are several studies that emphasize the importance of SEI on quality of life, and psychological health of people with chronic disease [24, 25]. This adverse effects was more among older adults, blacks and individuals with lower income or educational levels in Höfelmann et al. study [25].

Considering the importance of assessing the HRQL of adolescents as an indicator of justice in health and its impact on social and individual factors, the current study aims to analyze the role of socioeconomic inequalities in health-related quality of life (QoL) among Iranian young people in the middle stage of adolescence.

## Material & method

### Type of study & study setting

The cross-sectional study was conducted in Zanjan city, a province northwest of Iran from December 2020 to February 2021. The study setting included all public health centers that were located in the capital of the province. The study population included all young people in the middle stage of adolescence who were referred to the centers.

### Participants & sampling procedure

The total number of young people in the middle stage of adolescence that is covered by health community centers in Zanjan city is 17,213 people. The appropriate sample size given the population size and specified combination of precision and variability was calculated at 376 people by Cochran Formula. Also, considering the acceptable percentage of quality of life of 50% based on the study conducted by Arsanjani Shirazi et al. in 2015 [26], statistical error of 0.05, and acceptable accuracy of 0.04, the total sample size was estimated at 600 persons. The largest sample size was considered the final sample size.

Inclusion criteria were healthy adolescents, aged 15–18 years, and willingness to participate in the study. In Iran, all adolescents living in urban and rural areas, are covered by public health centers that provide primary health care based on Iranian service packages for children between 5 and 18 years. These centers are located in different parts of the city with different socioeconomic status. All clients can be identified by a national electronic identification code. There is no child-friendly service in Zanjan.

In this study, eligible adolescents were selected using the multistage sampling method in this way. Level 1: to access the diversity of socio-economic samples, the residential areas of Zanjan city were clustered into four-level based on municipal zoning. The city has 4 municipal zoning and 35 health centers. Level 2: three health centers were selected randomly in each selected area. Level 3: the eligible cases are randomly selected using their identification number through a national electronic identification system (CIB), and then data be collected through interviews by trained providers. In this way, they called the person and after explaining the objectives of the study and obtaining verbal consent to participate in the study, the questionnaires were completed according to the statements of the participants. If there are no eligible people in the household, who did not want to participate in the study or did not answer the phone number after two calls the selected number is removed and the next number replaced. The response rate in this study was about 75%.

### Measures

#### **Demographic questionnaire**

A self-designed questionnaire was used to assess socioeconomic and demographic characteristics including Age, Sex, Birth rank, Father’s job, Mother’s job, Father’s education, Mother’s education, Family Income, Family home ownership status, Insurance, and assets. To collect the household asset data, we used a questionnaire in which we asked if their family-owned any of the 16 household assets including “Microwave, Refrigerator, Freezer, Furniture, Computer, Laptop, Color TV, Landline, Washing Machine, Dish Washing Machine, Cell Phone, Stove with Oven, Steam Cleaner Wash Floor, Car, Handmade Carpets, and Vacuum Cleaner). The principal component analysis (PCA) was used to reduce the large data sets of asset ownership variables into a smaller number of dimensions. Because smaller data sets are easier and faster to analyze without extraneous variables to process. A total of 6 items (Laptop, Microwave, Washing Machine, Dish Washing Machine, Handmade Carpets, Steam Cleaner Wash) were retained with higher eigenvalues (> 1) accounting for 51.164% of the variance. Then household assets are categorized into quintiles (Poorest, Poorer, Middle, Richer, Richest).

We also collect a family’s income status on a qualitative scale at three levels” lower than adequacy”, “Adequate”, and” More than adequacy”.

#### The KIDSCREEN-52 questionnaire

The KIDSCREEN is a self-report measure to assess children’s and adolescents’ health-related quality of life (HRQOL) aged from 8 to 18 years. The time frame refers to the past week. The questionnaire was developed and tested within a European public health project [27]. The questionnaire included ten dimensions: Physical- (5 items), Psychological Well-being (6 items), Moods and Emotions (7 items), Self-Perception (5 items), Autonomy (5 items), Parent Relations and Home Life (6 items), Social Support and Peers (6 items), School Environment (6 items), Social Acceptance (Bullying) (3 items), Financial Resources (3 items). The scoring of each question is in a five-point of a Likert scale, with the frequency of a particular behavior or feeling (1 = never, 2 = rarely, 3 = sometimes, 4 = often, 5 = always) or the severity of attitude (1 = not at all, 2 = partially, Shows 3 = average, 4 = very much, 5 = infinite). The following questions are scored in reverse (12-13-14-15-16-17-18-21-22-23-50-51-52). The overall scores ranged from 52 to 260. A higher score means a better quality of life. In this study, the overall score of QOL was categorized in tertile levels. The score lower than 25%, 25 to 75%, and more than 75% of total scores were considered poor, moderate, and good levels, respectively.

The validity and reliability of this questionnaire was assessed for the current samples. A first-order model was used for confirmatory factor analysis (CFA). The fit indices in our model were 0.98, 0.96, 0.91, and 0.03 for CFI, TLI, GFI, RMSEA, respectively, therefore this model has good acceptance. Data analysis showed satisfactory internal consistency with Cronbach’s alpha ranging from 0.84 to 0.93 in all dimensions.

### Statistical analysis

Data weighted by the population covered for each municipal zoning. The data were analyzed using the Statistical Package for Social Science (SPSS) 16. Descriptive statistics including the mean ± SD, frequencies, and percentages used to describe the demographic status of participants. The independent t-test and chi-square test were also used to compare the continuous and dichotomous variables between the groups, respectively.

The Health Equity Assessment Toolkit (HEAT) Version 4.0 (beta) was used to assess adolescents’ QOL inequalities in terms of socio-economic subgroups. A summary measure of health inequality, including Concentration Index, Ratio (R), and Modified Gini was used to report numerical results. The Gini index < 0.2 represents perfect income equality, 0.2–0.3 relative equality, 0.3–0.4 adequate equality, 0.4–0.5 big income gap, and above 0.5 represents a severe income gap.

## Results

A total of 600 questionnaires were completed. Twenty-one questionnaires were not analyzed due to incomplete data; therefore, the reported results included 579 samples. The comparison of demographic characteristics of adolescents in term of gender have been shown in Table 1 according to the results, the highest percentage of participants in both groups had 17 years old. The comparison of age groups between male and female gender was shown a statically significant difference. Meanwhile, the comparison of birth rank, insurance, family income, her/his father’s and mother’s education, and the job status of her/his father and mother between the male and female gender was not significant (Table 1).

**Table 1:** The comparison of baseline socio-demographic characteristics in term of gender (*n* = 579) *

Variables		Frequency (%)		*P*-Value
		Female	Male	
Asset Component	Steam Cleaner Wash Floor (yes)	36(12.5)	46(15.8)	0.261
	Stove with Oven (yes)	97(33.8)	111(38.3)	0.263
	Handmade carpets (yes)	71(24.7)	85(29.2)	0.217
	Microwave oven (yes)	75(26.0)	85(29.2)	0.394
	Dish Washing Machine (yes)	51(17.8)	57(19.6)	0.575
	Laptop (yes)	113(39.2)	109(37.5)	0.660
Age(year)	15	91(31.6)	70(24.1)	0.046
	16	73(25.3)	62(21.3)	
	17	88(30.6)	115(39.5)	
	18	36(12.5)	44(15.1)	
Ownership Status	Personal	225(78.1)	245(84.2)	0.085
	Leased	57(19.8)	38(13.1)	
	Other	6(2.1)	8(2.7)	
Father’s job	Unemployed	28(9.9)	23(8.0)	0.523
	Worker	30(10.6)	35(12.2)	
	Self-employer	120(42.3)	109(38.0)	
	Government employee	66(23.2)	67(23.3)	
	Other	40(14.1)	53(18.5)	
Mother’s job	Unemployed	236(82.6)	228(79.7)	0.393
	Employed	50(17.5)	58(20.3)	
Mother’s education	Illiterate	11(3.8)	19(6.6)	0.245
	Primary	71(24.7)	61(21.1)	
	Guidance	56(19.4)	52(18)	
	High school& diploma	92(31.9)	83(28.7)	
	College	58(20.1)	74(25.6)	
Insurance	Yes	229(80.4)	239(82.4)	0.525
	No	56(19.6)	51(17.6)	
Birth rank	1	141(49.0)	138(47.6)	0.741
	> 1	147(51.0)	152(52.4)	
Family income	Lower than adequacy	191(66.6)	199(68.4)	0.492
	Adequate	82(28.6)	73(25.1)	
	More than adequacy	14(4.9)	19(6.5)	
Father’s education	Illiterate	14(4.9)	10(3.5)	0.095
	Primary	54(18.8)	40(13.9)	
	Guidance	43(15)	50(17.4)	
	High school& diploma	106(36.9)	93(32.3)	
	College	70(24.4)	95(33)	

### QOL status of participants

The mean (SD) and median QOL were 196.51(17.22) and 196.00 respectively. Furthermore, the percentage of poor, moderate, and good level of QOL was 27.2%, 49.8%, and 23% respectively.

### Socio-economic inequality

Considering the median cutoff point of the quality-of-life score, the socio-economic inequality, and the quality of life compared between male and female adolescents. The result showed that the asset, father’s education, mother’s education, and family income in female adolescents, the assets, and family income in male adolescents were significantly related factors to the quality of life (*p* < 0.05) (Table 2).

**Table 2:** Socio-economic inequality of QoL in term of gender

Variables		Lower Than Median		Upper Than Median		*P* Value
		Female N(%)	Male N(%)	Female N(%)	Male N(%)	
Asset Quantile	Poorest	40(26.5)	28(20.9)	20(16.0)	27(18.8)	Female = 0.045 Male = 0.021
	Poorer	28(18.5)	37(27.6)	19(15.2)	31(21.5)	
	Middle	39(25.8)	27(20.1)	29(23.2)	20(13.9)	
	Richer	25(16.6)	26(19.4)	28(22.4)	26(18.1)	
	Richest	19(12.6)	16(11.9)	29(23.2)	40(27.8)	
Father’s Job	Government Employee	30(19.4)	25(18.0)	38(27.9)	41(28.1)	Female = 0.08 Male = 0.062
	Self-Employer	62(40.0)	50(36.0)	58(45.0)	59(40.4)	
	Worker	22(14.2)	22(15.8)	8(6.2)	13(8.9)	
	Other	23(14.8)	27(19.4)	17(13.2)	25(17.1)	
	Unemployed	18(11.6)	15(10.8)	10(7.8)	8(5.5)	
Mother’s Job	Unemployed	132(84.1)	111(81.0)	104(80.6)	117(79.6)	Female = 0.444 Male = 0.762
	Employed	25(15.9)	26(19.0)	25(19.4)	30(20.4)	
Father’s Education	Illiterate	10(6.3)	4(2.9)	4(3.1)	6(4.1)	Female = 0.041 Male = 0.128
	Primary	35(22.2)	21(15.0)	19(14.7)	19(13.0)	
	Guidance	27(17.1)	31(22.1)	16(12.4)	19(13.0)	
	High School, Diploma	57(36.1)	47(33.6)	49(38.0)	46(31.5)	
	College	29(18.4)	37(26.4)	41(31.8)	56(38.4)	
Mother’s Education	Illiterate	9(5.7)	9(6.4)	2(1.5)	10(6.8)	Female = 0.006 Male = 0.118
	Primary	41(25.9)	36(25.7)	30(23.1)	25(17.0)	
	Guidance	39(24.7)	29(20.7)	17(13.1)	23(15.6)	
	High School, Diploma	39(24.7)	39(27.9)	53(40.8)	44(29.9)	
	College	30(19.0)	27(19.3)	28(21.5)	45(30.6)	
Family income	Lower than adequacy	120(75.9)	117(83.0)	71(55.0)	82(55.4)	Female = 0.001 Male = 0.001
	Adequate	34(21.5)	20(14.2)	48(37.2)	52(35.1)	
	More than adequacy	4(2.5)	4(2.8)	10(7.8)	14(9.5)	
Family home ownership status	Personal	117(74.1)	117(83.0)	108(83.1)	126(85.1)	Female = 0.117 Male = 0.211
	leased	36(22.8)	22(15.6)	21(16.2)	16(10.8)	
	other	5(3.2)	2(1.4)	1(0.8)	6(4.1)	

The comparison of quality-of-life component scores between male and female adolescents showed that the physical and autonomy components were significantly more in male versus school environment components scores in female adolescents (Table 3).

**Table 3:** The comparison of quality-of-life components between male and female

QOL components	Sex	Mean (SD)	*P* value
Physical	Female	18.62(3.00)	0.001
	Male	19.44(3.02)	
Psychological Well-being	Female	22.50(3.02)	0.984
	Male	22.51(3.26)	
Moods and Emotions	Female	29.47(3.61)	0.450
	Male	29.23(4.27)	
Self-Perception	Female	19.42(2.97)	0.786
	Male	19.35(2.90)	
Autonomy	Female	17.66(2.94)	0.036
	Male	18.17(2.96)	
Parent Relations and Home Life	Female	23.64(2.94)	0.120
	Male	23.26(3.00)	
Financial Resources	Female	10.54(2.08)	0.521
	Male	10.64(1.94)	
Social Support and Peers	Female	18.94(4.36)	0.979
	Male	18.93(4.38)	
School Environment	Female	21.99(2.91)	0.044
	Male	21.47(3.29)	
Social Acceptance (Bullying)	Female	13.71(1.38)	0.514
	Male	13.79(1.51)	
Total score	Female	179.20(14.40)	0.03
	Male	179.16(16.04)	

There were economic-related inequalities in the QOL of adolescents in Zanjan. The score of QOL increases with increasing economic status. QoL was lowest in the poorest quintile (40.3) and the highest in the richest quintile (65.7). The risk of lower QOL in the poorest quintile was 1.12 times more than in the richest quintile (Table 4). The consideration index of Asset in terms of sex was 4.5 and the modified Gini index was more than 0.5 in females and males. The result showed that the quality of life of both sexes was affected by economic status (Table 5).

**Table 4:** Economic- related inequality in QOL

Asset	affected population	Estimate	CI 95%		Setting average
Poorest	24.0%	40.3	32.0	48.5	47.9
Poorer	19.8	43.5	33.4	51.8	
Middle	19.8	43.5	34	52.7	
Richer	18.3	50.9	41.3	60.6	
Richest	18.1	65.7	56.5	74.9	

Risk Q1/Q5 = 24/19.8 = 1.12

**Table 5:** Consideration index of asset in term of sex

Index		N of obs.	Index value	Std. error	*P* value
Modified Gini	Female	276	0.61403232	0.00097112	0.001
	Male	280	0.61981277	0.00132458	0.001
Absolute concentration index			4.5	-	

## Discussion

The current study aims to analyze the role of socioeconomic inequalities in health-related quality of life (QoL) among Iranian young people in the middle stage of adolescence. Our results show that the majority of participants had moderate levels of HRQL. The component of HRQL was different between female and male adolescents. In males, physical and autonomy components were more than in females, and school environment components had more effect on the HRQL of female adolescents. Moreover, there are positive correlations between socio-economic backgrounds and HRQL.

Many studies highlight the importance of socioeconomic inequality on the adolescent quality of life [5, 15, 17, 20]. In this regard, Hovsepian et al. study reported a positive association between socioeconomic status, school functioning, psychosocial function, and total score of HRQOL in both males, and female adolescents. Moreover, in their study, all components of HRQL except its social subscale were low in female adolescents, [28] while in our study, boy adolescents only have better scores in the physical and autonomy components of HRQL than girls.

The field of autonomy examines the adolescent’s freedom of choice, self-sufficiency, and independence, and considers the individual’s opportunities to create leisure and social activity [29]. Upon entering this period of life, very profound biological, psychological and social changes occur in adolescents [1, 30]. The need for connection can create a conflict between the desires of the adolescent and their family, thereby putting his or her independence at risk [30]. We observed a lower score of autonomy component in girls, which indicated more communication problems with parents. It seems that socio-cultural issues related to gender role, preference and more attention for the male sex, brain development, and different physiological changes following puberty play an important role in this difference.

The school environment was one of the main components of HRQL and has a greater impact on girls vs. boys. Similar to our results, Gaspar et al. show that female adolescents compared with males had a lower level of quality of life in all domains except school environments [31]. In the same sense, another study revealed that adolescents with positive perceptions about their school climate had good self-rated health, school satisfaction, and quality of life [31].

Explaining the role of gender differences in health-related quality of life, Hyde states in his gender similarity hypothesis that males and females are similar in most psychological variables. Only in variables such as motor behavior, aggression, and some aspects of sexual activity, there are differences between the two sexes [32]. Furthermore, physical changes during puberty in girls, such as the onset of menstruation and hormonal imbalances, decrease their psychological well-being compared to boys [33]. We also observe those female adolescents compared with males had lower scores in the physical component. In this regard, Michel state that physical changes during puberty and conflict with exaggerated cultural norms of beauty make girls feel more unbalanced in their physical and mental well-being [34].

In this study, the relationship between income inequality and HRQL was evaluated using the Concentration Index, Ratio (R), and Modified Gini Index. The Gini index as one of the measures of health inequality examines the distribution of income, or in other words, the distribution of wealth among a population. The Gini coefficient is a number between zero and 1(0 denoting complete equality and 1 complete inequality). We observed a modified Gini index of more than 0.5 in females and males, which indicated a severe income gap. Assessing the relationship between these variables, and HRQL reveal that the quality of life in both sexes was affected by economic status. Our finding also reveals that socioeconomic variables that affect HRQL are different between male and female adolescents. Parental education was one of the main factors of female adolescents, and variables such as assets, and family income were effective on HRQL of both groups. These results are consistent with the findings of other studies in this field [5, 15, 17, 20]. In line with our study, Spurrier et al. reported a high level of HRQL among children in families of higher income, educated, as well as employed. Moreover, they reported living in an original family with both parents has a positive impact on HRQL [35]. Also, in Barriuso-Lapresa, et al. study, there was a high quality of life, and mental health for children of mothers who had a university degree, and high social classes. [36] The results of one path analysis show the major effects of socioeconomic status (e.g., occupational prestige, household income, and parental education), social support on HRQL, and health behavior such as smoking, and toothbrush in adolescents [37]. Another study demonstrated that gender, ethnicity, maternal education level, socio-economic status, and weight status are the main predictors of HRQL [38].

Our study was conducted during the second peak of the COVID-19 pandemic. Previous literature has documented the negative effects of this pandemic on both HRQL and family income [39–42]. Studies have shown that the COVID-19 outbreaks have caused the biggest shock to the world economy, so the implementation of government disease control policies, such as social exclusion and quarantine, has led to the temporary closure of businesses [39, 40]. Shrinking the global economy, increasing unemployment, poverty, income inequality, gender inequality, and widening the gap between different countries are just some effects of this pandemic [40, 41]. All of these factors can have adverse effects on a person’s quality of life. Tran et al. study show a negative effect of COVID-19 on household income and psychological health. And they state that having a chronic disease, female gender, and living in a family with 3–5 members were related to low-quality life [43]. In another study, during the outbreak, socio-economic inequality was one of the main predictors of death in Brazilian children [44]. Ravens-Sieberer et al. reported that children and adolescents experienced a high level of anxiety, mental health problems, and low HRQL after the COVID-19 pandemic, and socioeconomic status, migration background, and limited living space had the greatest impact [45]. In Adıbelli et al. study, the score of the quality of life of children was good, but their parents reported that in their children gained weight, tendency to sleep and internet use increased during the pandemic [46].

Around the world, evidence shows that people with poorer socioeconomic status suffer from lower levels of health, and quality of life. Many of these inequalities, which are the result of socio-economic differences between various groups of people, are unfair and unjust. In any country, along with individual and family factors, the cultural and political factors governing that country, as well as, corruption, and economic interests play an important role in socio-economic inequalities [47]. Therefore, it is not possible to plan to reduce inequalities without considering the role of governments.

### Limitation

One of the limitations of this study is that we didn’t assess the influence of other variables such as psychological wellbeing, parental relationship, and COVID-19-related issues, which can affect the HRQL of adolescents. It has been suggested that future studies consider them in the study design. However, this study was conducted during the QOVID-19 pandemic, but their information (such as disease status, the impact of COVID-19 on their socioeconomic status, quarantine, etc.) is not available. Also, the design of this study is cross-sectional, so the causal relationship between the variables is not predictable.

## Conclusion

Our findings highlight the significant effects of socioeconomic inequality on the HRQL of Iranian adolescents. Since having a healthy society requires improving the quality of life, it is suggested that health policymakers consider comprehensive planning in the field of economic equality, and improving the quality of life of adolescents. Socio-economic inequalities should also be considered when designing interventions to improve adolescents’ quality of life.

## Data Availability

The data sets used and analyzed during the current study are available from the corresponding author upon reasonable request.
